# HIV Preexposure Prophylaxis Awareness and Referral to Providers Among Hispanic/Latino Persons — United States, 2019

**DOI:** 10.15585/mmwr.mm7040a1

**Published:** 2021-10-08

**Authors:** Shubha Rao, Mesfin S. Mulatu, Mingjing Xia, Guoshen Wang, Wei Song, Aba Essuon, Deesha Patel, Adanze Eke, Emilio J. German, MSHSA

**Affiliations:** 1 Division of HIV Prevention, National Center for HIV, Viral Hepatitis, STD, and TB Prevention, CDC.

Hispanic or Latino* (Hispanic) persons are disproportionately affected by HIV in the United States. In 2019, Hispanic persons accounted for 18% of the U.S. population, but for 29% of new diagnoses of HIV infection (*1*). The Ending the HIV Epidemic in the U.S. (EHE) initiative aims to reduce new HIV infections by 90% by 2030 (*2*). Preexposure prophylaxis (PrEP), medication taken to prevent acquisition of HIV, is an effective strategy for preventing HIV infection.^†^ To examine PrEP awareness and referral to providers among Hispanic persons, CDC analyzed 2019 National HIV Prevention Program Monitoring and Evaluation HIV testing data. Approximately one quarter (27%) of Hispanic persons tested for HIV at CDC-funded sites (n = 310,954) were aware of PrEP, and 22% of those who received a negative HIV test result and were eligible for referral (111,644) were referred to PrEP providers. PrEP awareness and referrals among Hispanic persons were lower compared with those among non-Hispanic White persons. Among Hispanic persons, significant differences were found in PrEP awareness and referrals by age, gender, race, population group, geographic region, and test setting. HIV testing programs can expand PrEP services for Hispanic persons by implementing culturally and linguistically appropriate strategies that routinize PrEP education and referral, collaborating with health care and other providers, and addressing social and structural barriers.

CDC analyzed 2019 National HIV Prevention Program Monitoring and Evaluation HIV testing data submitted by 60 CDC-funded state, local, and territorial health departments^§^ and 29 directly funded community-based organizations to assess measures of PrEP awareness^¶^ and referral to a PrEP provider.** Persons whose HIV status is negative are eligible for PrEP referral when they meet the clinical criteria for PrEP prescription based on CDC guidelines or local protocols. PrEP awareness among persons tested for HIV infection was defined by an affirmative response documented by HIV test providers to the question, “Has the client ever heard of PrEP?” Similarly, PrEP referral among persons eligible for referral was defined by an affirmative response documented by HIV test providers to the question, “Was the client given a referral to a PrEP provider?” PrEP awareness and referrals among Hispanic persons were compared with those of persons of other racial and ethnic groups. PrEP measures among Hispanic persons were also compared by age, gender, race,^††^ ethnicity,^§§^ test setting, ^¶¶^ U.S. Census region,*** and population groups defined by transmission risk.^†††^ Robust Poisson regression was used to calculate prevalence ratios (PRs) and 95% confidence intervals (CIs). This activity was reviewed and approved by CDC and conducted consistent with applicable federal law and CDC policy.^§§§^

During 2019 in the United States, 2,341,342 CDC-funded HIV tests were conducted. These included 546,337 (23.3%) tests conducted among Hispanic persons, 919,066 (39.3%) among non-Hispanic Black/African American (Black) persons, 658,496 (28.1%) among non-Hispanic White (White) persons, and 217,443 (9.3%) among persons of other or unspecified race. Among all tested persons with PrEP-related data, PrEP awareness was slightly higher among Hispanic persons (27.4%) than among Black persons (26.2%; PR = 1.05; 95% CI = 1.04–1.06) but lower than that among White persons (31.4%; PR = 0.87; 95% CI = 0.87–0.88) and those of other racial or ethnic groups (42.1%; PR = 0.65; 95% CI = 0.64–0.66) (Figure).

**FIGURE:**
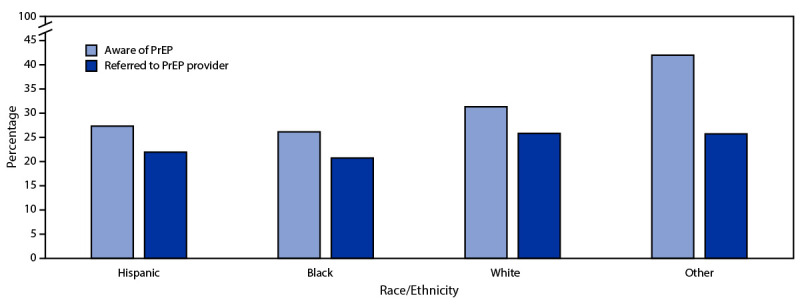
Preexposure prophylaxis awareness and referral to preexposure prophylaxis providers, by race and ethnicity* — United States, 2019^†^^,^^§^ **Abbreviation:** PrEP = preexposure prophylaxis. * Black, White, and persons of other races were non-Hispanic; Hispanic persons could be of any race. ^†^ Valid HIV tests for this analysis include tests for which a test result (i.e., positive or negative) was known and had a nonmissing value for PrEP awareness and referral. ^§^ PrEP awareness among persons tested for HIV infection was defined by an affirmative response documented by HIV test providers to the question, “Has the client ever heard of PrEP?” PrEP referral among persons eligible for referral was defined by an affirmative response documented by HIV test providers to the question, “Was the client given a referral to a PrEP provider?” This analysis excluded HIV tests with missing values on PrEP awareness and referral to a PrEP provider.

Among Hispanic persons, awareness was higher among persons aged 25–49 years (29.9%; PR = 1.07) and lower among those aged ≥50 years (17.0%; PR = 0.60) compared with those aged 13–24 years (28.1%) (Table 1). By gender, compared with females, 14.5% of whom were aware of PrEP, awareness was highest among transgender persons (68.6%; PR = 4.74) followed by males (36.6%; PR = 2.53). Awareness was higher among Black Hispanic persons (39.3%; PR = 1.91) and persons of other races (39.3%; PR = 1.91) than among White Hispanic persons (20.6%). Compared with heterosexual Hispanic females (awareness = 17.5%), PrEP awareness was higher among gay, bisexual, and other men who have sex with men (MSM) (63.5%; PR = 3.62), persons who inject drugs (28.9%; PR = 1.65), and heterosexual males (21.5%; PR = 1.22). Awareness was higher among persons tested in non–health care settings (35.4%; PR = 1.95) than among those tested in health care settings (18.1%). By U.S. Census region, PrEP awareness was lower among Hispanic persons tested in the West (49.1%; PR = 0.87), Midwest (30.1%; PR = 0.54), South (13.4%; PR = 0.24), and U.S. territories (12.9%; PR = 0.23) than among those tested in the Northeast (56.2%).

**TABLE 1:** Preexposure prophylaxis awareness among Hispanic persons tested for HIV infection, by demographic characteristics, U.S. Census region, and test setting — United States, 2019

Characteristic	No. of persons (column %)		PR (95% CI)
	Tested for HIV infection*	Aware of PrEP*	
**Total (row %)**	**310,954 (100.0)**	**85,288 (27.4)**	**N/A**
**Age group, yrs^†^**			
13–24	80,166 (25.8)	22,494 (28.1)	Ref
25–49	183,396 (59.0)	54,887 (29.9)	1.07 (1.05–1.08)
≥50	44,226 (14.2)	7,500 (17.0)	0.60 (0.59–0.62)
**Gender** ^§^			
Female	133,308 (42.9)	19,308 (14.5)	Ref
Male	172,769 (55.6)	63,207 (36.6)	2.53 (2.49–2.56)
Transgender	3,517 (1.1)	2,414 (68.6)	4.74 (4.62–4.86)
**Race** ^¶^			
White	185,173 (59.5)	38,181 (20.6)	Ref
Black	20,488 (6.6)	8,054 (39.3)	1.91 (1.87–1.94)
Other	10,110 (3.3)	3,978 (39.3)	1.91 (1.86–1.96)
Population group**			
Heterosexual female	88,234 (28.4)	15,469 (17.5)	Ref
Gay, bisexual, and other male who has sex with males	66,657 (21.4)	42,312 (63.5)	3.62 (3.57–3.68)
Person who injects drugs	11,937 (3.8)	3,444 (28.9)	1.65 (1.59–1.70)
Heterosexual male	65,276 (21.0)	14,010 (21.5)	1.22 (1.20–1.25)
**Test setting** ^††^			
Health care setting	181,348 (58.3)	32,846 (18.1)	Ref
Non–health care settings	109,231 (35.1)	38,637 (35.4)	1.95 (1.93–1.98)
**U.S. Census region**			
Northeast	32,232 (10.4)	18,109 (56.2)	Ref
Midwest	17,139 (5.5)	5,159 (30.1)	0.54 (0.52–0.55)
South	173,218 (55.7)	23,259 (13.4)	0.24 (0.24–0.24)
West	75,479 (24.3)	37,095 (49.1)	0.87 (0.86–0.89)
U.S. territories^§§^	12,886 (4.1)	1,666 (12.9)	0.23 (0.22–0.24)

**Abbreviations:** CI = confidence interval; N/A = not applicable; PR = prevalence ratio; PrEP = preexposure prophylaxis; Ref = referent group.

* Valid HIV tests for this analysis included tests for which a test result (i.e., positive or negative) was known and had a nonmissing value on PrEP awareness. PrEP awareness was assessed by HIV test providers documenting a response to the following question, ”Has the client ever heard of PrEP?”

^†^ For age, the numbers of records missing or invalid are as follows: 3,166 (1.0%) in the column “Tested for HIV infection” and 407 (0.5%) in the column “Aware of PrEP.”

^§^ For gender, the numbers of records missing or invalid are as follows: 1,360 (0.4%) in the column “Tested for HIV infection” and 359 (0.4%) in the column “Aware of PrEP.”

^¶^ Race categories include the following: “White” = Hispanic White; “Black” = Hispanic Black or African American; and “Other” = Hispanic persons of other races including Asian, American Indian or Alaska Native, Native Hawaiian or Other Pacific Islander, and multirace. For race, the numbers of records missing or invalid are as follows: 95,183 (30.6%) in the column “Persons tested for HIV infection” and 35,075 (41.1%) in the column “Aware of PrEP.”

** For population groups, the numbers of records missing or invalid are as follows: 23,002 (7.4%) in the column “Tested for HIV infection” and 3,399 (4.0%) in the column “Aware of PrEP.” In addition, the numbers of records for “other” excluded from this table are as follows: 55,848 (18.0%) in the column “Tested for HIV infection” and 6,654 (7.8%) in the column “Aware of PrEP.”

^††^ Mobile settings and unknown settings are excluded.

^§§ ^Includes Puerto Rico and the U.S. Virgin Islands.

Overall, referral to a PrEP provider was higher among Hispanic persons (22.0%) compared with non-Hispanic Black persons (20.8%; PR = 1.06; 95% CI = 1.04–1.07) but lower when compared with non-Hispanic White persons (25.9%; PR = 0.85; 95% CI = 0.84–0.86) and those of other racial/ethnic groups (25.8%; PR = 0.85; 95% CI= 0.83–0.87) (Figure).

Among Hispanic persons eligible for referral to a PrEP provider, PrEP referral was higher among Hispanic persons aged 25–49 years (22.8%; PR = 1.05) and lower among those aged ≥50 years (16.6%; PR = 0.77) compared with those aged 13–24 years (21.7%) (Table 2). By gender, referral was higher among transgender persons (30.3%; PR = 2.04) and males (25.7%; PR = 1.73) than among females (14.8%). PrEP referral was lower among Black Hispanic persons (13.4%; PR = 0.55) and Hispanic persons of other races (21.6%; PR = 0.89) than among White Hispanic persons (24.3%). PrEP referral was higher among Hispanic MSM (39.5%; PR = 2.57) and persons who inject drugs (17.2%; PR = 1.12) but lower among heterosexual males (11.7%; PR = 0.76) than heterosexual females (15.4%). By test setting, PrEP referral was lower among persons tested in non–health care settings (20.4%; PR = 0.83) than among those tested in health care settings (24.6%). By U.S. Census region, PrEP referral was higher among Hispanic persons tested in the Midwest (32.9%; PR = 2.14), South (26.9%; PR = 1.75), and West (17.8%; PR = 1.16) and lower among those tested in U.S. territories (13.4%; PR = 0.87) compared with persons tested in the Northeast (15.4%).

**TABLE 2:** Referral to preexposure prophylaxis providers among Hispanic persons who were eligible for PrEP, by demographic characteristics, U.S. Census region, and test setting — United States, 2019

Characteristic	Eligible for a PrEP referral*	Referred to a PrEP provider*	PR (95% CI)
	no. (column %)	no. (row %)	
**Total**	**111,644 (100.0)**	**24,506 (22.0)**	**N/A**
**Age group, yrs^†^**			
13–24	32,698 (29.3)	7,088 (21.7)	Ref
25–49	68,061 (61.0)	15,538 (22.8)	1.05 (1.03–1.08)
≥50	10,333 (9.3)	1,717 (16.6)	0.77 (0.73–0.80)
**Gender** ^§^			
Female	39,339 (35.2)	5,828 (14.8)	Ref
Male	69,966 (62.7)	17,981 (25.7)	1.73 (1.69–1.78)
Transgender	1,920 (1.7)	581 (30.3)	2.04 (1.90–2.19)
**Race** ^¶^			
White	58,960 (52.8)	14,318 (24.3)	Ref
Black	11,235 (10.1)	1,509 (13.4)	0.55 (0.53–0.58)
Other	4,795 (4.3)	1,037 (21.6)	0.89 (0.84–0.94)
Population group**			
Heterosexual females	32,429 (29.0)	4,980 (15.4)	Ref
Gay, bisexual, and other male who has sex with males	34,583 (31.0)	13,645 (39.5)	2.57 (2.50–2.64)
Person who injects drugs	6,777 (6.1)	1,166 (17.2)	1.12 (1.06–1.19)
Heterosexual male	27,814 (24.9)	3,243 (11.7)	0.76 (0.73–0.79)
**Test setting** ^††^			
Health care settings	54,105 (48.5)	13,323 (24.6)	Ref
Non–health care settings	53,574 (48.0)	10,916 (20.4)	0.83 (0.81–0.85)
**U.S. Census region**			
Northeast	28,325 (25.4)	4,353 (15.4)	Ref
Midwest	8,445 (7.6)	2,775 (32.9)	2.14 (2.05–2.23)
South	45,878 (41.1)	12,363 (26.9)	1.75 (1.70–1.81)
West	25,450 (22.8)	4,540 (17.8)	1.16 (1.12–1.21)
U.S. territories^§§^	3,546 (3.2)	475 (13.4)	0.87 (0.80–0.95)

**Abbreviations:** CI = confidence interval; N/A = not applicable; PR = prevalence ratio; PrEP = preexposure prophylaxis; Ref = referent group.

* Eligibility for a PrEP referral was assessed by HIV test providers documenting a response to the question, ”Was the client eligible for a referral to a PrEP provider?” Referral to a PrEP provider was assessed by HIV test providers documenting a response to the question, “Was the client given a referral to a PrEP provider?” HIV tests with missing values for eligibility for PrEP referral and referral to a PrEP provider were excluded.

^†^ For age, the numbers of records missing or invalid are as follows: 552 (0.5%) in the column “Eligible for a PrEP referral” and 163 (0.7%) in the column “Referred to a PrEP provider.”

^§^ For gender, the numbers of records missing or invalid are as follows: 419 (0.4%) in the column “Eligible for a PrEP referral” and 116 (0.5%) in the column “Referred to a PrEP provider.”

^¶^ Race categories include the following: “White” = Hispanic White; “Black” = Hispanic Black or African American; and “Other” = Hispanic persons of other races including Asian, American Indian or Alaska Native, Native Hawaiian or Other Pacific Islander, and multirace. For race, the numbers of records missing or invalid are as follows: 36,654 (32.8%) in the column “Eligible for a PrEP referral” and 7,642 (31.2%) in the column “Referred to a PrEP provider.”

** For population groups, the numbers of records missing or invalid are as follows: 1,747 (1.6%) in the column “Eligible for a PrEP referral,” and 360 (1.5%) in the column “Referred to a PrEP provider.” In addition, the numbers of records for “other” excluded from this table are as follows: 8,294 (7.4%) in the column “Eligible for a PrEP referral” and 1,112 (4.5%) in the column “Referred to a PrEP provider.”

^††^ Mobile settings and setting unknown are excluded.

^§§ ^Includes Puerto Rico and the U.S. Virgin Islands.

## Discussion

Approximately one in four Hispanic persons who received a CDC-funded HIV test was aware of PrEP, and approximately one in five who were eligible for PrEP referral was referred to a PrEP provider. PrEP use is increasing among Hispanic persons in the United States (*3*); however, low levels of PrEP awareness and referrals to PrEP providers among Hispanic persons in general and compared with non-Hispanic White persons suggest a need to identify and remove barriers to awareness of, referral to, and receipt of PrEP services. Routinizing PrEP education and referrals, expanding coverage for PrEP medications, and implementing culturally and linguistically relevant strategies might improve optimal and equitable use of PrEP among Hispanic persons at risk for HIV infection (*4*).

PrEP awareness and referral were higher among Hispanic MSM and transgender persons than among those in other population groups. This finding is consistent with other studies that have documented higher PrEP coverage among MSM and transgender persons (*3*,*5*). Given that HIV incidence and prevalence are substantially higher among MSM and transgender persons (*1*,*6*), efforts to further increase PrEP awareness and referral among these populations are important to reach persons who might benefit from a PrEP prescription. PrEP referral was lower among Black Hispanic persons compared with that among White Hispanic persons, consistent with lower PrEP coverage among Black persons compared with other racial or ethnic groups (*6*), suggesting that Black Hispanic persons might experience additional challenges to accessing PrEP services.

Hispanic persons tested in the South and U.S. territories had the lowest levels of PrEP awareness. Communities in the South and U.S. territories are disproportionately affected by HIV (*2*,*6*) and have higher need for PrEP services. Low PrEP coverage in the South and other regions is attributed to individual, social, and structural barriers, including lack of health insurance; PrEP- and HIV-related stigma; lower HIV risk perception; limited availability of PrEP services in primary care and sexually transmitted disease clinics and community health centers; and lack of effective messaging about PrEP (*7*–*9*). In addition, immigration status, English language fluency, and education level are barriers to PrEP access among Hispanic persons (*8*).

PrEP referrals were higher among Hispanic persons tested in health care settings than among those tested in non–health care settings. Health care settings might have routinized referrals to PrEP providers. Health care providers can improve PrEP awareness and use by discussing PrEP benefits, developing culturally tailored messages to destigmatize PrEP, and integrating PrEP into routine primary care (*7*,*9*). Establishing linkage agreements with clinical providers and expanding PrEP navigation might increase PrEP referrals in non–health care settings (*10*).

The findings in this report are subject to at least three limitations. First, data were based on CDC-funded HIV testing programs that were not representative of all U.S. HIV testing programs or persons receiving PrEP care in non–CDC-funded HIV testing programs. Second, data were collected at the test level and might overrepresent persons tested multiple times. Finally, the percentages of Hispanic persons who were aware of PrEP and those referred to a PrEP provider might be overestimated because missing records were excluded from the denominators.

Broader implementation of PrEP services among Hispanic persons at risk for HIV infection is an essential strategy of the EHE initiative (*2*). CDC has developed an integrated HIV prevention campaign, Let’s Stop HIV Together/Detengamos Juntos el VIH, ^¶¶¶^ featuring messaging and resources to increase PrEP awareness and use among Spanish speakers. In addition, the Ready, Set, PrEP**** program provides free PrEP medication to eligible persons. HIV prevention programs can help achieve the goals of the EHE initiative by addressing individual, social, and structural barriers to receipt of PrEP services, collaborating with health care and other providers, expanding health care coverage, and implementing culturally and linguistically relevant strategies for Hispanic persons.

**Summary**

**What is already known about this topic?**

Hispanic or Latino (Hispanic) persons are disproportionately affected by HIV. Preexposure prophylaxis (PrEP) is an effective strategy to prevent HIV infection.

**What is added by this report?**

Approximately one in four Hispanic persons tested for HIV at CDC-funded sites was aware of PrEP, and 22% of those eligible for referral were referred to PrEP providers. PrEP awareness and referrals among Hispanic persons were lower compared with those among non-Hispanic White persons.

**What are the implications for public health practice?**

HIV testing programs can expand PrEP services for Hispanic persons by implementing culturally and linguistically appropriate strategies that routinize PrEP education and referral, collaborating with health care and other providers, and addressing social and structural barriers.

## Footnotes

* Hispanic persons can be of any race.

†
https://www.cdc.gov/hiv/pdf/risk/prep/cdc-hiv-prep-guidelines-2017.pdf

§ Fifty states, the District of Columbia, Puerto Rico, the U.S. Virgin Islands, and seven Metropolitan Statistical Areas or specified Metropolitan Divisions: Baltimore, Chicago, Houston, Los Angeles County, New York City, Philadelphia, and San Francisco.

¶ PrEP awareness was assessed by HIV test providers documenting a response to the question, “Has the client ever heard of PrEP (PreExposure Prophylaxis)?” The PrEP awareness question was required from all persons testing for HIV although the response could have been collected before or after the test was performed.

** Referral to PrEP providers was assessed by HIV test providers documenting a response to the question, “Was the client given a referral to a PrEP provider?” Referral was provided if the person testing negative for HIV infection met the appropriate clinical criteria for using PrEP or was determined to be eligible for a PrEP referral based on CDC guidelines or local protocol. Referral to providers in this report might include passive referral (e.g., client is provided information about the PrEP provider) as well as active referral (e.g., client is assisted with contacting and making an appointment with the PrEP provider).

†† Race refers to the client's self-reported classification or classifications of the biologic heritage with which they most closely identify. For this report, Hispanic persons are stratified into three race groups: Black or African American (Black), White, and other, which includes American Indian or Alaska Native, Asian, Native Hawaiian or Other Pacific Islander persons, or those with more than one race.

§§ Ethnicity refers to the client's self-report of whether they are of Hispanic or Latino origin.

¶¶ For each CDC-funded test, test setting is the location where the test was administered. Health care settings are clinical settings in which both medical diagnostic and treatment services were provided (e.g., primary care clinics, community health centers, emergency departments). Non–health care settings are nonclinical settings in which neither medical diagnostic nor treatment services are provided (e.g., schools or educational facilities, faith-based facilities, and field testing sites).

*** State and local health department jurisdictions were categorized into the four U.S. Census regions (Northeast, Midwest, South, and West) (https://www2.census.gov/geo/pdfs/maps-data/maps/reference/us_regdiv.pdf). Puerto Rico and the U.S. Virgin Islands were grouped into the category “U.S. territories.”

††† Data on behavioral risk characteristics were reported for the last 5 years before the HIV test. Men who have sex with men (MSM) includes males who reported male-to-male sexual contact and represents gay, bisexual, and other MSM; and males who reported both male-to-male sexual contact and injection drug use. Persons who inject drugs include persons who reported injection drug use. Heterosexual males include males who reported only heterosexual contact with a female. Heterosexual females include females who reported only heterosexual contact with a male. Others include transgender persons who inject drugs, transgender persons, women who have sex with women, men or women who have sex with transgender persons, and persons with no history of sexual contact or injection drug use.

§§§ 45 C.F.R. part 46, 21 C.F.R. part 56; 42 U.S.C. Sect. 241(d); 5 U.S.C. Sect. 552a; 44 U.S.C. Sect. 3501 et seq.

¶¶¶ https://www.cdc.gov/stophivtogether/index.html?CDC_AA_refVal=https%3A%2F%2Fwww.cdc.gov%2Factagainstaids%2Fabout%2Findex.html

****
https://www.hiv.gov/federal-response/ending-the-hiv-epidemic/prep-program
